# A Certain Form of Muscular Atrophy Peculiar to the Lower Extremities

**Published:** 1882-10

**Authors:** 


### A CERTAIN FORM OF MUSCULAR ATROPHY PECULIAR TO THE LOWER EXTREMITIES.

The large class of amyotrophies from medullar origin consists of two distinct groups: the deuteropathic and the protopathic atrophies. Progressive muscular atrophy, with all its variations, forms one group of this class; it is also called the Aran-Duchesne type. The irregular march of this disease, its affecting the upper part of the body, and especially the arms and hands, distinguish this class. But there is a muscular atrophy, made known by Dr. Friedreich, which is characterized by its etiology and course of symptoms. This atrophy is a hereditary disease, and it attacks only the lower extremities and hips. It is the same disease known among English physicians as the Weatherbee ail, four generations of the Weatherbee family having been afflicted with the disease. Eulenburg reports a case of three sisters, who were taken sick when eight years of age, and who suffered from deformities of the severest character, caused by this atrophy. Bernhard has seen five brothers suffering from this disease. Naunyn reports cases of a family where the disease has been hereditary for six generations. In the hospital Hotel-Dieu, at Paris, there is at the present time a patient showing the symptoms of the disease in a most peculiar form. He is twenty-six years of age; a hackman; the muscles of the upper part of the body strongly developed. When eighteen years of age, he felt a weakness in the region of the kidneys, and he could but with difficulty climb upon the seat of his hack. The course of the disease has been very slow, and in the last three months the weakness in the limbs has increased to a considerable degree. The muscles in the upper part of the body are very vigorous, and in contrast with that, the muscles on the lower extremities have partially or entirely disappeared. The mass of sacro-lumbar muscles, the extensors and adductors of the upper part of the limb, have atrophied to such a degree that the humerus can be felt as only covered by the skin. The biceps, semi-tendinous and semi-membranous, are regular. The triceps femoralis contracts with good effect. The appearance is that of pseudo-hypertrophic paralysis, and the gait of the patient is the same as in those affected with this disease. The patient walks, balancing laterally, the body resting on one or the other side. By the preservation of the triceps femoralis and the atrophy of the extensors, the patient walks on the anterior part of his feet, there being veritable equinism. The patient can walk with a cane, but he is tired in a very short time. The difficulty in sacending steps is extreme, and he does it mostly by going on hands and feet. There is no trouble from any of the sphincter muscles, as in other ataxic diseases. The atrophied muscles do not act when electricity is applied.—*France Médicale.*

